# Supplementary material for: Bitesize bundles F-actin and influences actin remodeling in syncytial Drosophila embryo development
Source: bioRxiv. 2025 Jun 21:2023.04.17.537198. Originally published 2023 Apr 17. Preprint. [Version 2] doi: 10.1101/2023.04.17.537198 (PMC10153138; doi:10.1101/2023.04.17.537198)
Supplement: Supplement 5 [file NIHPP2023.04.17.537198v2-supplement-5.pdf]

bioRxiv preprint doi: https://doi.org/10.1101/2023.04.17.537198; this version posted June 21, 2025. The copyright holder for this preprint (which was not certified by peer review) is the author/funder, who has granted bioRxiv a license to display the preprint in perpetuity. It is made available under aCC-BY 4.0 International license.

Supplemental Figure 1

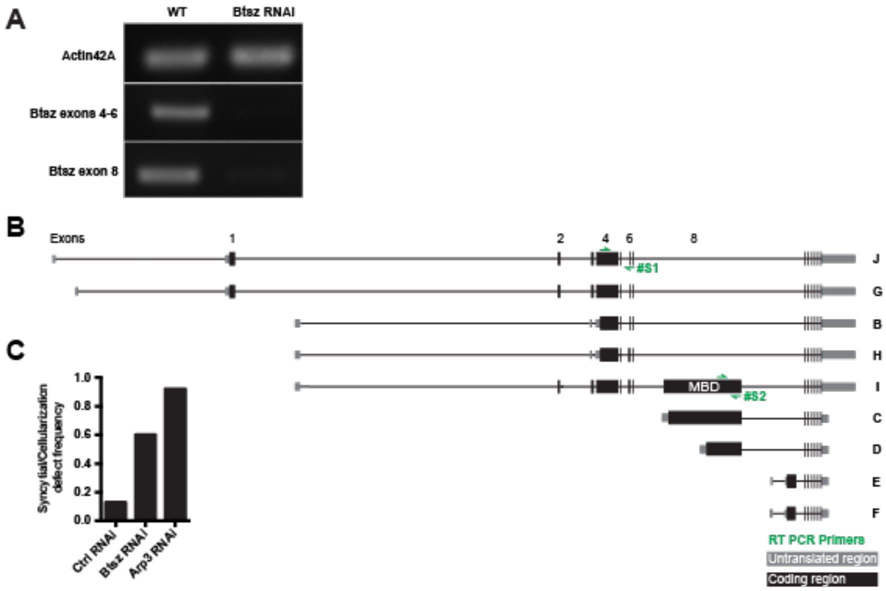

Supplemental Figure 1. (A) RT-PCR Gel of Btsz cDNA showing maternal knockdown of Btsz in the early embryo. Primers used are shown in S1B. Primer pair S1 was used for Btsz exons 4-6 and pair S2 was used for Btsz exon 8, the MBD. **(B)** Schematic of the nine splice isoforms of the Btsz protein. Letters on the right-hand side denote the isoform name. Location of RT-PCR primer pairs #S1 and #S2 for exons 4-6 and exon 8, respectively, are shown with green arrows. **(C)** Frequency of control RNAi, Btsz RNAi, and Arp3 RNAi embryos with defects during the syncytial or cellularization stages. 4 out

bioRxiv preprint doi: https://doi.org/10.1101/2023.04.17.537198; this version posted June 21, 2025. The copyright holder for this preprint (which was not certified by peer review) is the author/funder, who has granted bioRxiv a license to display the preprint in perpetuity. It is made available under aCC-BY 4.0 International license.

of 30 control, 25 out of 42 Btsz RNAi, and 12 out of 13 Arp3 RNAi embryos displayed defects.

bioRxiv preprint doi: https://doi.org/10.1101/2023.04.17.537198; this version posted June 21, 2025. The copyright holder for this preprint (which was not certified by peer review) is the author/funder, who has granted bioRxiv a license to display the preprint in perpetuity. It is made available under aCC-BY 4.0 International license.

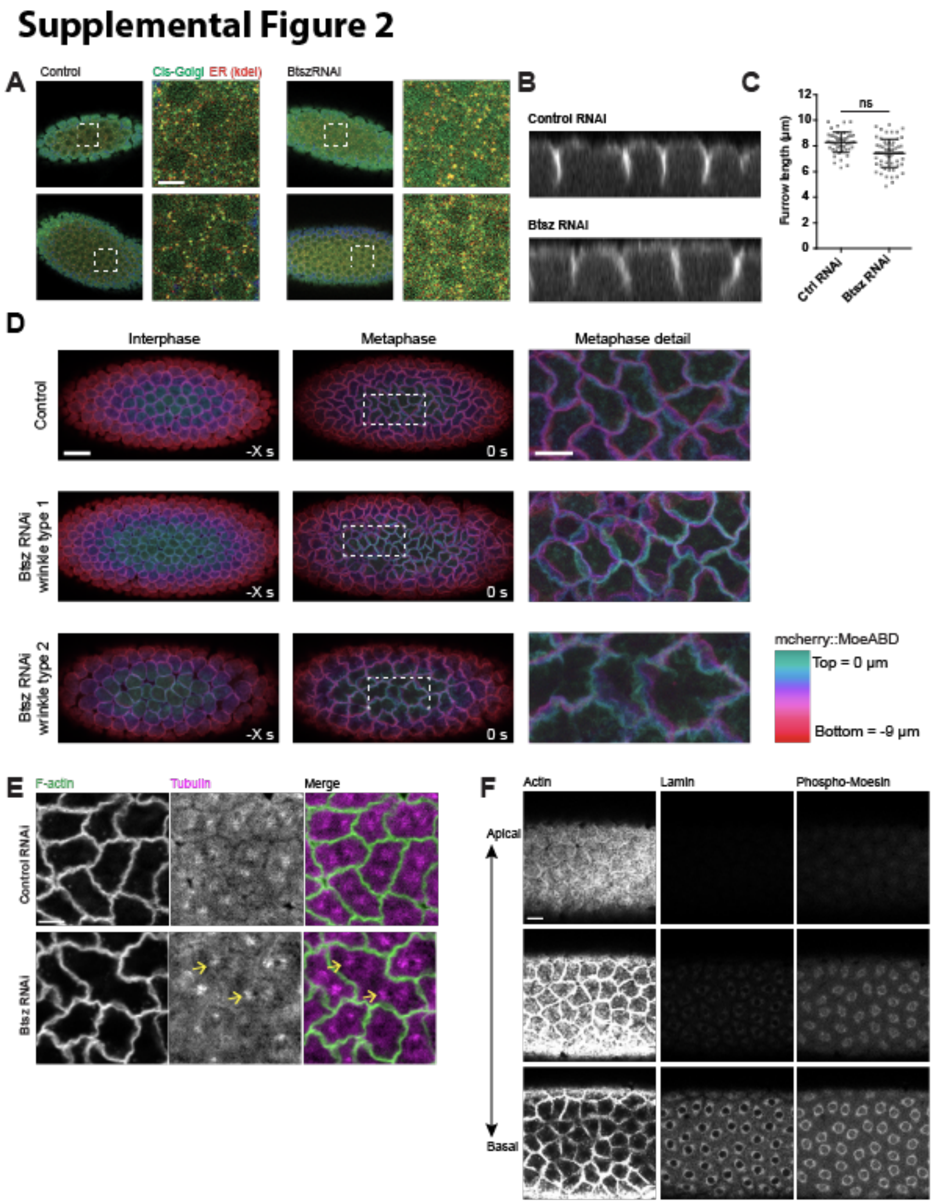

Supplemental Figure 2. (A) Maximum intensity projections of cortical grazing slices of control and Btsz-RNAi embryos stained for ER (anti-KDEL) and Cis-golgi (anti-gm130). Morphology and overlap between stained compartments were not disrupted in Btsz-RNAi. Scale bar = 5 µm. **(B)** Cross section of pseudo-cleavage furrows during syncytial blastoderm development. Actin was visualized live with the mCherry::MoesinABD marker. **(C)** Pseudo-cleavage furrows that form normally in Btsz RNAi embryos are not significantly different in length compared to the wildtype. Each point is one furrow from n = 6 embryos for both control RNAi and Btsz RNAi. **(D)** Intensity projection of mCherry::MoesinABD with different depths encoded by color (right, colormap). Control embryos have tight distribution of colors, while Btsz-RNAi embryos often have

bioRxiv preprint doi: https://doi.org/10.1101/2023.04.17.537198; this version posted June 21, 2025. The copyright holder for this preprint (which was not certified by peer review) is the author/funder, who has granted bioRxiv a license to display the preprint in perpetuity. It is made available under aCC-BY 4.0 International license.

dispersed color signals indicating tilted or wavy pseudo-cleavage furrow morphology. **(E)** Slice of fixed wildtype and Btsz RNAi embryos during metaphase. F-actin was visualized using Phalloidin and tubulin was visualized using an α-tubulin antibody. Pseudo-cleavage furrows that have receded lead to two sets of spindles in one compartment (yellow arrows). Scale bar is 5 µm. **(F)** Grazing sections of the F-actin network (Phalloidin), Lamin B, and phospho-Moesin in the wild-type syncytium at metaphase at dfferent depths. Phospho-Moesin localizes to perinuclear regions, closely overlapping with Lamin B and is absent from F-actin caps and pseudo-cleavage furrows.

bioRxiv preprint doi: https://doi.org/10.1101/2023.04.17.537198; this version posted June 21, 2025. The copyright holder for this preprint (which was not certified by peer review) is the author/funder, who has granted bioRxiv a license to display the preprint in perpetuity. It is made available under aCC-BY 4.0 International license.

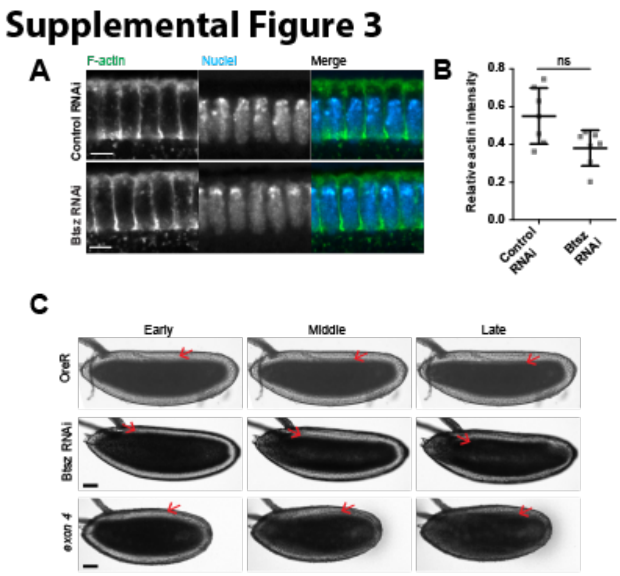

Supplemental Figure 3 (A) Cross section of fixed control RNAi or Btsz-RNAi embryos during cellularization. F-actin was visualized using Phalloidin and nuclei were visualized using Hoechst. Scale bar = 5μm. **(B)** There is no significant difference between the relative F-actin intensity (ratio of actin intensity in the apical region to that of the furrow canals) in the wildtype and Btsz-RNAi embryos. Mann-Whitney U test. **(C)** Brightfield images of control, btsz-RNAi, and btsz[exon4] mutant embryos undergoing cellularization. Red arrows indicate cellularization front. Note that cellularization front is often uneven in Btsz-RNAi and btsz mutant embryos, likely due to nuclear fallout and density defects.

bioRxiv preprint doi: https://doi.org/10.1101/2023.04.17.537198; this version posted June 21, 2025. The copyright holder for this preprint (which was not certified by peer review) is the author/funder, who has granted bioRxiv a license to display the preprint in perpetuity. It is made available under aCC-BY 4.0 International license.

Supplemental Figure 4

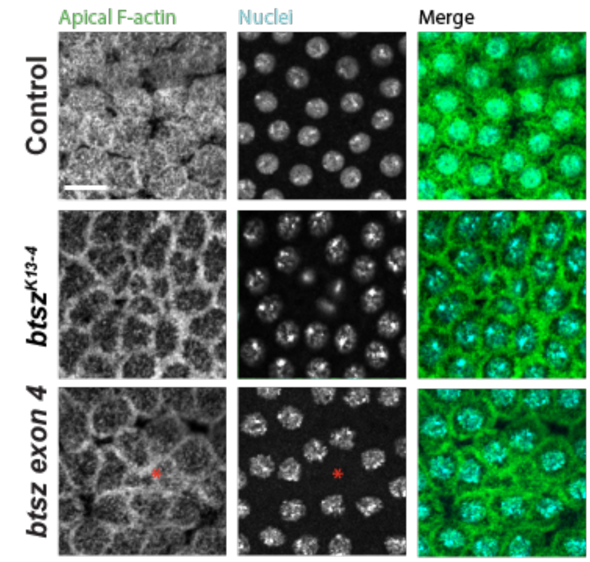

Supplemental Figure 4. Btsz mutants have syncytial embryos defects. Images are grazing sections showing cortical F-actin (phalloidin staining) and nuclei (Hoechst staining) in control and btsz[k13-4] and btsz[exon4] mutants. Note that mutants exhibit regions that lack nuclei under actin caps (red asterisk), similar to the nuclear fallout phenotype of Btsz-RNAi. Scale bar = 10 μm.
